# Novel Strategy to Control Transgene Expression Mediated by a Sendai Virus-Based Vector Using a Nonstructural C Protein and Endogenous MicroRNAs

**DOI:** 10.1371/journal.pone.0164720

**Published:** 2016-10-20

**Authors:** Masayuki Sano, Minoru Iijima, Manami Ohtaka, Mahito Nakanishi

**Affiliations:** Biotechnology Research Institute for Drug Discovery, National Institute of Advanced Industrial Science and Technology (AIST), Tsukuba, Ibaraki, Japan; University of North Carolina at Chapel Hill, UNITED STATES

## Abstract

Tissue-specific control of gene expression is an invaluable tool for studying various biological processes and medical applications. Efficient regulatory systems have been utilized to control transgene expression in various types of DNA viral or integrating viral vectors. However, existing regulatory systems are difficult to transfer into negative-strand RNA virus vector platforms because of significant differences in their transcriptional machineries. In this study, we developed a novel strategy for regulating transgene expression mediated by a cytoplasmic RNA vector based on a replication-defective and persistent Sendai virus (SeVdp). Because of the capacity of Sendai virus (SeV) nonstructural C proteins to specifically inhibit viral RNA synthesis, overexpression of C protein significantly reduced transgene expression mediated by SeVdp vectors. We found that SeV C overexpression concomitantly reduced SeVdp mRNA levels and genomic RNA synthesis. To control C expression, target sequences for an endogenous microRNA were incorporated into the 3′ untranslated region of the *C* genes. Incorporation of target sequences for miR-21 into the SeVdp vector restored transgene expression in HeLa cells by decreasing C expression. Furthermore, the SeVdp vector containing target sequences for let-7a enabled cell-specific control of transgene expression in human fibroblasts and induced pluripotent stem cells. Our findings demonstrate that SeV C can be used as an effective regulator for controlling transgene expression. This strategy will contribute to efficient and less toxic SeVdp-mediated gene transfer in various biological applications.

## Introduction

Efficient gene delivery systems are in demand in modern biology and clinical settings. Although the efficacy and safety of various non-viral vectors has improved [1], recombinant viral vectors are primarily used in gene therapy clinical trials because of their transduction efficiency, broad tropisms, and long-term gene expression [2, 3]. Stable and persistent gene expression is crucial for long-term supplementation of therapeutic genes and is typically achieved by integrating transgenes into the host chromosome. Recently, integrating vectors, such as retroviral and lentiviral vectors, have been successfully used to create induced pluripotent stem (iPS) cells [4, 5]. Human iPS (hiPS) cells have received increasing attention in regenerative and molecular medicine owing to their proliferative and developmental potential. However, because chromosomal insertion of transgenes may cause tumor formation [6], alternative approaches that can reduce the risk of tumorigenesis while maintaining long-term gene expression should be considered for clinical applications.

Sendai virus (SeV) is a nonsegmented negative-strand RNA virus, a member of the *Paramyxovirus* genus in the *Paramyxovirinae* subfamily [7]. SeV harbors a single-strand RNA genome encoding six main proteins: nucleocapsid protein (NP), phosphoprotein (P), matrix protein (M), glycoproteins (F and HN), and large protein (L). SeV can infect a large variety of animal cells; however, it is neither pathogenic nor carcinogenic in humans. Thus, various applications have been explored to employ SeV as a recombinant viral vector in medical research and clinical trials [8]. Recently, SeV-based vectors have been used as a superior gene delivery system to create iPS cells [9, 10]. We developed a unique delivery/expression system using a replication-defective and persistent SeV (SeVdp) vector based on a noncytopathic variant SeV strain, Cl.151 [10, 11]. SeVdp vectors accommodate and stably express multiple exogenous genes in infected cells [10, 12]. In this system, the replication and transcription of SeVdp occur entirely in the cytoplasm, and long-term transgene expression is sustained without chromosomal insertion. Importantly, blocking SeV replication using short interfering RNA (siRNA) against the polymerase *L* gene can completely erase the genomic RNA from infected cells [10]. We demonstrated that an SeVdp vector harboring *Oct4*, *Sox2*, *Klf4*, and *c-Myc* genes efficiently reprogrammed somatic cells into transgene-free iPS cells [10, 13, 14].

Although SeVdp vectors are suitable for long-term transgene expression, overexpression of transgenes in undesired cells or tissues may cause adverse effects. Efficient regulatory systems, including the tetracycline-inducible system, have been used to control transgene expression in various types of DNA viral or integrating viral vectors [15]. These systems provide efficacy and safety, reducing the side effects attributed to unwanted gene expression. Although an SeVdp vector equipped with a potent gene regulatory system is very attractive for controlling transgene expression in a specific cell type, it is difficult to transfer these existing regulatory systems to an SeV vector platform. Unlike DNA promoter-based gene expression systems that depend on the host transcriptional machinery, SeV encodes an RNA-dependent RNA polymerase (RdRp) composed of the P and L proteins, and the replication and transcription of SeV completely depend on RdRp activity [16]. RdRp initiates viral RNA synthesis by recognizing *cis*-acting elements placed on the negative (-)-strand genomic and positive (+)-strand antigenomic RNAs. A leader (Le) sequence at the 3′ end of the genomic RNA acts as a promoter to direct the antigenome synthesis, whereas the antigenomic RNA possesses the trailer (Tr) promoter at its 3′ end for genome synthesis [7]. In addition, (-)-strand genomic RNA contains gene-start and gene-end signals at each gene boundary [17], which are recognized by RdRp to synthesize viral monocistronic mRNAs. These *cis*-acting elements are highly conserved and tightly coupled with RdRp-mediated RNA synthesis. Currently, there is lack of data for the detailed analysis of binding mode of RdRp to these *cis*-acting elements. Thus, a system for controlling transgene expression for SeV-based vectors has not been reported.

In the present study, we focused on SeV nonstructural C proteins for regulating transgene expression mediated by SeVdp vectors. The C proteins are a nested set of four proteins, C′, C, Y1, and Y2, and are translated from P mRNA; however, they are initiated at different start sites [18, 19]. Although previous studies have revealed that C proteins have multiple functions [20–23], one of their key roles is regulating viral RNA synthesis. It was demonstrated that the C proteins specifically inhibit viral transcription as well as replication [24–26]. Recently, it was suggested that C proteins play a role in controlling the levels of genomic and antigenomic RNAs through promoter-specific inhibition of viral replication [27]. Based on these findings, we hypothesized that the SeV C protein could be used as an effective regulator to control transgene expression mediated by SeVdp vectors. We found that SeV C overexpression effectively inhibited SeVdp RNA synthesis, reducing transgene expression levels.

To control transgene expression, we attempted to regulate C expression depending on endogenous microRNA (miRNA) activities. miRNAs, a class of small regulatory RNAs, modulate gene expression via translational repression or mRNA degradation by binding to the 3′ untranslated regions (3′ UTR) of target mRNAs [28]. Many miRNAs exhibit distinct expression patterns in different cell types and play crucial roles in various biological processes, including metabolism, development, and differentiation [29, 30]. Recent studies demonstrated that endogenous miRNAs could be exploited to regulate transgene expression in various types of vectors. For example, the lentiviral vector containing target sequences for hematopoietic-enriched miR-142-3p at the 3′ UTR of the transgene escaped immune-mediated vector clearance in mice, resulting in prolonged and stable transgene expression [31]. Incorporation of target sequences for liver-specific miR-122 into adeno-associated viral vectors restricted transgene expression in non-liver cells [32, 33]. miRNA-regulated systems would be widely applicable in the control of transgene expression in target cells [34]. We demonstrate here that regulation of C expression by an endogenous miRNA enables the SeVdp vector to control transgene expression in a cell-specific manner. This strategy is expected to make the SeVdp vector a more versatile tool in various biological and medical applications.

## Materials and Methods

### Plasmid construction

cDNA encoding puromycin-N-acetyltransferase (puromycin resistance protein: Puro^r^) or human codon-optimized C (C-opt) was synthesized by GenScript. cDNA encoding Puro^r^ or C-opt was inserted between the BsrGI and XhoI sites of the pON1 plasmid, which contains a cytomegalovirus (CMV) immediate-early enhancer/promoter and zeocin resistance gene (*Zeo*^*r*^), yielding pCMV-Pur or pCMV-C-opt, respectively. The pON1 plasmid was constructed by replacing *Puro*^*r*^ to *Zeo*^*r*^ and by changing a multiple cloning site on the pIRESpuro vector (Clontech). cDNA encoding P or L was inserted into the pJOI11 plasmid to yield pCMV-P or pCMV-L, respectively. The pJOI11 was constructed by inserting the sequences for CMV immediate-early enhancer/promoter, mini intron, SV40 late poly(A) signal, and multiple cloning site into the pGEM5zf (+) vector (Promega). pCMV-P contains mutations in the *P* gene to abolish C and V expression. To prepare the reporter plasmids psi-miR-21, psi-let-7a, and psi-miR-scr, the hybridized sense (5′-TCGATCAACATCAGTCTGATAAGCTA-3′), (5′-TCGAAACTATACAACCTACTACCTCA-3′) or (5′-TCGAGACAGCTAATCTCGTAACATAT-3′) and antisense (5′- GGCCTAGCTTATCAGACTGATGTTGA-3′), (5′-GGCCTGAGGTAGTAGGTTGTATAGTT-3′) or (5′-GGCCATATGTTACGAGATTAGCTGTC-3′) strands were inserted between the XhoI and NotI sites of the psiCHECK-2 plasmid (Promega), respectively. The scramble sequence was predicted using the siRNA Wizard v3.1 software (InvivoGen). To prepare the HaloTag-fused C-opt expression plasmid, cDNA encoding C-opt was inserted between the SgfI and PmeI sites of the pFN28A HaloTag CMV-neo Flexi vector (Promega).

### Cell culture and transfection

HeLa S3 cells [35], BHK/T7/151M(SE) cells [10] and normal human dermal fibroblasts (NHDF; KURABO) were cultured in Dulbecco’s modified Eagle’s medium (Sigma-Aldrich) supplemented with 10% fetal bovine serum and penicillin-streptomycin (Wako). HEK293 [36] and LLC-MK_2_ [37] cells were cultured in Minimum Essential Medium Eagle (Sigma-Aldrich) supplemented with 10% fetal bovine serum and penicillin-streptomycin. Human iPS cells were established from TIG-3 cells [36] with the SeVdp vector harboring *Oct4*, *Sox2*, *Klf4*, and *c-Myc* genes, and were cultured in mTeSR1 medium (STEMCELL Technologies) on an iMatrix-511-coated plate (Nippi). For transfection of pCMV-Pur and pCMV-C, 1.5 × 10^5^ of HeLa S3 cells harboring the SeVdp vector were seeded into a 12-well plate and transfected with 1.0 μg of the plasmid using Lipofectamine 2000 reagent according to the manufacturer’s instructions (Thermo Fisher Scientific). For transfection of pCMV-P and pCMV-L, 1.5 × 10^5^ of HeLa S3 cells harboring the SeVdp vector were seeded into a 12-well plate and transfected with 1.0 μg of either plasmid or 0.5 μg of both plasmids using Lipofectamine 2000 reagent. For transfection of anti-C-opt siRNA, 1.0 × 10^5^ of HeLa S3 cells harboring the SeVdp vector were seeded into a 24-well plate and transfected with 50 nM of siRNA using the Lipofectamine RNAi MAX reagent (Thermo Fisher Scientific). Anti-C-opt siRNA (sense: 5′-ACGAGGAGGUGCAUCCCUATT-3′, antisense: 5′- UAGGGAUGCACCUCCUCGUTT-3′) and negative control siRNA were purchased from Nippon Gene. To determine the activity of anti-C-opt siRNA, 5.0 × 10^4^ of HeLa S3 cells were seeded into a 24-well plate and co-transfected with the anti-C-opt siRNA and 500 ng of the HaloTag-fused C-opt plasmid using Lipofectamine 2000 reagent. Two days after transfection, the cells were stained with the HaloTag TMR ligand (Promega) and DAPI (Vector Laboratories) according to the manufacturer’s instructions. For the miRNA inhibition assay, 5.0 × 10^4^ of HeLa S3 cells harboring the SeVdp vector were seeded into a 24-well plate and transfected with 50 nM of the miR-21 seed-targeting 8-mer locked nucleic acid (LNA) oligonucleotide (antimiR-21) [38] or the LNA scramble [38] using Lipofectamine RNAi MAX reagent. The LNA oligonucleotides were synthesized by GeneDesign.

### Luciferase assay

To determine miR-21 activity, 8.0 × 10^4^ of HeLa S3 cells were seeded into a 24-well plate and transfected with 50 ng of the psi-miR-21 or psi-miR-scr plasmid using Lipofectamine 2000 reagent. To determine let-7 activity, 6.0 × 10^4^ of NHDF cells or 1.2 × 10^5^ of hiPS cells were seeded into a 24-well plate and transfected with 200 ng of the psi-let-7a or psi-miR-scr plasmid. Twenty-four hours after transfection, the activities of the firefly and *Renilla* luciferase were analyzed using the Dual-Luciferase Reporter Assay System (Promega). Firefly luciferase activities of BHK/T7/151M(SE) cells harboring the SeVdp vector were determined by luciferase reporter assay and normalized by protein concentration measured using the Pierce 660nm Protein Assay Reagent (Thermo Fisher Scientific).

### SeVdp vector production and infection

The SeVdp genome cDNA was constructed as previously described [10]. cDNAs encoding enhanced green fluorescent protein (EGFP) and firefly luciferase (Luc2CP) were amplified by PCR using pEGFP-1 (Takara Bio) and pGL4.12[luc2CP] (Promega) as templates, respectively. cDNA encoding hygromycin B phosphotransferase (hygromycin resistance protein: Hyg^r^) was synthesized by GenScript. cDNA encoding Puro^r^ or C-opt was prepared as described above. These cDNA fragments were used to construct vector cDNAs of SeV-EGFP, SeV-Pur, SeV-Pur2, SeV-C, SeV-C2, SeV-Pur2-Luc, and SeV-C2-Luc. To construct SeV-C2-21T and SeV-C2-let7T, four copies of the miR-21 complementary sequence (5′-TCAACATCAGTCTGATAAGCTACAGAATTCAACATCAGTCTGATAAGCTAATCAGATCAACATCAGTCTGATAAGCTATCCATTTCAACATCAGTCTGATAAGCTA-3′) and four copies of the let-7a complementary sequence (5′- AACTATACAACCTACTACCTCACAGAATAACTATACAACCTACTACCTCAATCAGAAACTATACAACCTACTACCTCATCCATTAACTATACAACCTACTACCTCA-3′) were inserted into the 3′ UTR of the *C-opt* genes, respectively.

To establish vector-packaging cells, cDNA plasmids (2 μg) and expression vector plasmids for SeV proteins (NP, P, Fmut, HN, and L) (1 μg each) and for furin (20 ng) were transfected into BHK/T7/151M(SE) cells using Lipofectamine LTX Plus reagent (Thermo Fisher Scientific), followed by treatment with hygromycin B at 100 μg/mL. The SeVdp vector was rescued by transient transfection of plasmids for M, Fmut and HN proteins in vector-packaging cells and was recovered from the culture supernatant after incubation at 32°C for 4 days. The supernatant was filtered through 0.45 μm cellulose acetate filters and stored at -80°C. Titers of SeVdp vectors were determined by immunostaining using an anti-NP antibody [10] after infection of the diluted SeVdp vector suspension into LLC-MK_2_ cells. Target cells were infected with the SeVdp vector at a multiplicity of infection of 3 for 4 h.

### Quantitative real-time PCR

Total RNA was extracted using ISOGEN reagent (Nippon Gene) and residual DNA was digested with the DNase I (Nippon Gene). cDNAs were synthesized using 500 ng of total RNA, 2.5 μM oligo(dT)_20_ primers, and the Superscript III First-Strand Synthesis System (Thermo Fisher Scientific) in a 20 μL reaction. mRNA levels were determined by quantitative real-time PCR (qPCR) using the SsoAdvanced Universal SYBR Green Supermix (Bio-Rad) and the following primers: *EGFP* (Fwd: 5′-AGAACGGCATCAAGGTGAAC-3′, Rev: 5′- TGCTCAGGTAGTGGTTGTCG-3′), *Puro*^*r*^ (Fwd: 5′-GTGCCTGCTTTTCTGGAGAC-3′, Rev: 5′-CCGGGTCATACACCATGTTC-3′), *NP* (Fwd: 5′-ATGCAGCAGTACGTCACAGG-3′, Rev: 5′-AGGCACTGCTGATCTTCGAT-3′), *P*/*C*/*V* (Fwd: 5′- GCAAGACGTGCCCTAAAGTC-3′, Rev: 5′-TTCCACGCTCTCTTGGATCT-3′), *L* (Fwd: 5′-TTCCCTGACCAGAAGTTTGAA-3′, Rev: 5′-TCCTGATTTCACGGGATGAT-3′), *C-opt* (Fwd: 5′-TGGACCATGGAAGAGACTCC-3′, Rev: 5′-CCCCTGATGAGAGTCCTCAA-3′), *GAPDH* (Fwd: 5′- CTTTGGTATCGTGGAAGGACTC-3′, Rev: 5′- GTAGAGGCAGGGATGATGTTCT-3′). To measure the level of genomic RNA, cDNA was synthesized under the same conditions described above except using the primer (5′-AGAACGGCATCAAGGTGAAC-3′), corresponding to nucleotide positions 476–495 of the *EGFP* gene. qPCR analysis was performed using the SsoAdvanced Universal SYBR Green Supermix and the following primers (Fwd: 5’-AGAACGGCATCAAGGTGAAC-3’, Rev: 5′-TGCTCAGGTAGTGGTTGTCG-3′). *GAPDH* expression was used as an internal control and for normalization of the PCR data.

### Western blot analysis

HeLa S3 cells harboring the SeVdp vector were lysed with lysis buffer [10 mM Tris-HCl, pH 7.8, 0.15 M NaCl, 1 mM EDTA, 1% NP-40, and the Protease Inhibitor Cocktail (Nacalai Tesque)] and rotated for 1 h at 4°C. Protein samples were separated by SDS-PAGE and transferred to a polyvinylidene fluoride (PVDF) membrane using the Trans-Blot Turbo Transfer System (Bio-Rad). The membrane was blocked with the SuperBlock T20 (TBS) blocking buffer (Thermo Fisher Scientific) and then incubated with rabbit polyclonal anti-NP antibody (0.3 μg/mL) for 1 h. The membrane was washed and incubated with the stabilized peroxidase conjugated goat anti-rabbit (H+L) (1:3000 dilution; Thermo Scientific) for 1 h. Bands were visualized using SuperSignal West Dura Extended Duration Substrate according to the manufacturer’s instructions (Thermo Fisher Scientific). The antibodies were removed using Restore Plus Western Blot Stripping Buffer (Thermo Fisher Scientific) and then incubated with a mouse monoclonal anti-ß-Actin antibody (1:1000 dilution; Medical & Biological Laboratories) for 1 h. Bands were detected in the same manner as was conducted for NP detection, except using the stabilized peroxidase conjugated goat anti-mouse (H+L) (1:3000 dilution; Thermo Scientific). To measure the level of HaloTag-fused C-opt protein, 4.0 × 10^5^ of HeLa S3 cells were seeded into a 6-well plate and cotransfected with 2.0 μg of pCMV-HaloTag-fused C-opt plasmid and 50 nM of siRNA (anti-C siRNA or negative control siRNA) using Lipofectamine2000. Protein samples were separated by SDS-PAGE and transferred to a PVDF membrane, and bands were detected using the anti-HaloTag monoclonal antibody (1:1000 dilution; Promega) and rabbit polyclonal anti-ß-Actin antibody (1:1000 dilution; Medical & Biological Laboratories), respectively.

### Cell proliferation assay

To evaluate cell proliferation, 1.0 × 10^3^ of HeLa S3 cells harboring the SeVdp vector were seeded into a 96-well plate. The absorbance at OD_570_ (optical density at 570 nm) was measured using CellQuanti-MTT Cell Viability Assay Kits according to the manufacturer’s instructions (BioAssay Systems).

### Flow cytometry analyses

The fluorescence signal of EGFP was measured using a Gallios flow cytometer (Beckman Coulter), and the mean fluorescence intensity was calculated using Kaluza software (Beckman Coulter). For intracellular SeV antigen staining, cells were transfected with 40 nM siRNA against the *L* gene [10] or negative control siRNA on the same day when 1.2 × 10^5^ of cells were seeded into a 12-well plate (day 0). The cells were re-seeded on days 3, 6, and 9, and transfected with both siRNAs on day 6. Thirteen days after the first transfection, cells were fixed with the FIX & PERM cell fixation and permeabilization kit reagent A (Thermo Fisher Scientific). The cells were resuspended in reagent B containing an anti-SeV NP monoclonal mouse antibody (3.0 μg/mL) [10] and incubated for 1 h. Subsequently, the cells were incubated with Alexa Fluor 647-conjugated secondary antibody (Thermo Fisher Scientific) and analyzed using the Gallios flow cytometer.

## Results

### SeV C overexpression attenuates transgene expression mediated by SeVdp vectors

**Fig 1:**
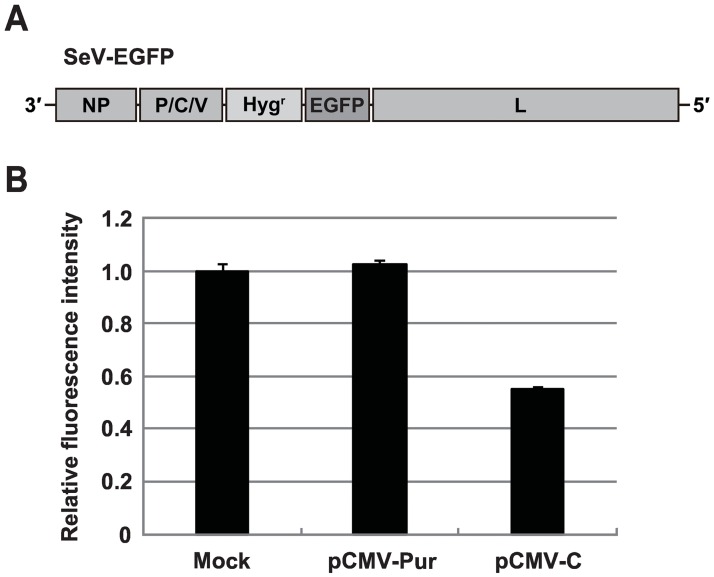
Inhibitory effect of SeV C on transgene expression mediated by the SeVdp vector. (A) Genome structure of SeV-EGFP. The *NP*, *P*, and *L* genes are indispensable for genome replication and transcription. The *P* gene contains multiple open reading frames encoding the P, C, and V proteins. Hygromycin resistance gene (*Hyg*^*r*^) and *EGFP* genes were inserted into the SeVdp backbone as transgenes. (B) Inhibition of transgene expression by SeV C overexpression. The plasmid containing either the human codon-optimized C gene (pCMV-C) or puromycin resistance gene (pCMV-Pur) under control of the CMV enhancer/promoter was transfected into HeLa S3 cells harboring the SeV-EGFP, and the levels of EGFP expression were measured by flow cytometry. The EGFP intensity of cells treated with transfection reagent (mock) was set to 1.0, and relative fluorescence intensities are indicated. The means and standard deviations (SD) from three replicate experiments are presented.

We initially constructed the SeVdp vector, SeV-EGFP encoding Hyg^r^, and EGFP to monitor transgene expression (Fig 1A). HeLa S3 cells were infected with SeV-EGFP, followed by treatment with hygromycin B to obtain the cells stably harboring the SeV-EGFP genome (SeV-EGFP cells). Persistent replication and transcription of the SeV-EGFP allowed long-term expression of EGFP. To examine whether SeV C overexpression could suppress transgene expression in SeV-EGFP cells, we constructed the plasmid vector pCMV-C, which contains the human codon-optimized SeV C (*C-opt*) gene under control of the CMV enhancer/promoter. SeV-EGFP cells were transfected with pCMV-C, and the level of EGFP expression was measured by flow cytometry 3 days after transfection. As shown in Fig 1B, pCMV-C reduced fluorescence intensity by 45% compared to the mock control, whereas pCMV-Pur, which encodes the Puro^r^, had no effect. This result indicates that SeV C overexpression attenuates transgene expression mediated by the SeVdp vector.

**Fig 2:**
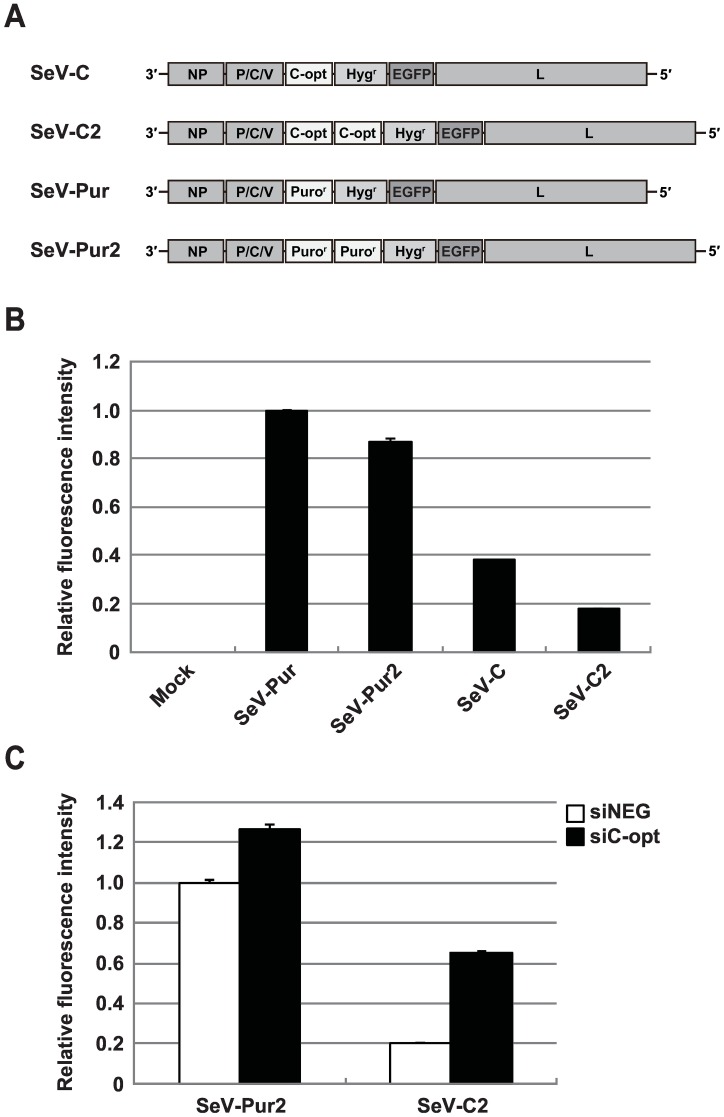
Effect of SeVdp-mediated C overexpression on transgene expression. (A) Genome structure of a series of SeVdp vectors. C-opt: human codon-optimized C gene, Puro^r^: puromycin resistance gene. (B) Inhibition of transgene expression by SeVdp-mediated C overexpression. HeLa S3 cells were infected with different SeVdp vectors and treated with hygromycin B. The levels of EGFP expression were measured by flow cytometry. EGFP intensity in SeV-Pur cells was set to 1.0, and relative fluorescence intensities of infected cells are indicated. Non-infected cells (mock) were used as a negative control. The means and SD from three replicate experiments are presented. (C) Effect of siRNA against the *C-opt* gene on transgene expression mediated by SeVdp vectors. Negative control siRNA (siNEG) or siRNA against the *C-opt* gene (siC-opt) was transfected into SeV-Pur2 or SeV-C2 cells. The levels of EGFP expression were measured by flow cytometry. The EGFP intensity of SeV-Pur2 cells transfected with the siNEG was set to 1.0, and relative fluorescence intensities of all cells are indicated. The means and SD from three replicate experiments are presented.

Next, we constructed the SeVdp vector, named SeV-C, containing the *C-opt* gene, followed by the *Hyg*^*r*^ and *EGFP* genes in the same vector backbone (Fig 2A). To increase C expression, the SeVdp vector containing two *C-opt* genes (SeV-C2) was also constructed. Because each *C-opt* gene is separately transcribed as a monocistronic mRNA by SeV RdRp, we expected that the SeV-C2 would produce high levels of C proteins compared to those produced by SeV-C. As controls, the SeV-Pur and SeV-Pur2, which contain *Puro*^*r*^ gene(s), were constructed. Notably, the *C-opt* and *Puro*^*r*^ genes are nearly identical in nucleotide length (*C-opt*; 618 bp, *Puro*^*r*^; 600 bp). The SeV-Pur and SeV-Pur2 were expected to be good controls to normalize EGFP expression in SeV-C and SeV-C2, respectively, because we found that the length and number of genes inserted into SeVdp markedly influenced downstream gene expression (data not shown). HeLa S3 cells were infected with SeV-C, SeV-C2, SeV-Pur, or SeV-Pur2 and EGFP expression was compared by fluorescence microscopy and flow cytometry after selection with hygromycin B (S1A and S1B Fig). Quantitative analysis based on flow cytometry indicated that in SeV-C and SeV-C2 cells, EGFP fluorescence was decreased by 62% and 82%, respectively, compared to in SeV-Pur cells, whereas SeV-Pur2 cells maintained EGFP expression (Fig 2B and S1B Fig). This result suggests that SeVdp vectors expressing relatively high levels of C protein gave rise to a strong reduction in transgene expression. These effects were also observed in HEK293 and LLC-MK_2_ cells (S2A Fig). We obtained similar results using SeV-C2-Luc and SeV-Pur2-Luc, which contain the firefly luciferase gene rather than the *EGFP* gene (S2B Fig).

To further confirm whether the reduction in EGFP fluorescence intensity was attributed to the SeV C overexpression, we used siRNA against the *C-opt* gene (siC-opt) to decrease the level of C protein. siC-opt showed strong inhibitory activity against C protein expression when siC-opt and a plasmid containing the HaloTag-fused *C-opt* gene under control of the CMV enhancer/promoter were co-transfected into HeLa S3 cells (S3A and S3B Fig). SeV-Pur2 or SeV-C2 cells were transfected with siC-opt, and the levels of EGFP expression were measured by flow cytometry 2 days after transfection (Fig 2C). siC-opt conferred a 3.2-fold increase in the fluorescence intensity compared to negative control siRNA (siNEG) in SeV-C2 cells, indicating that SeV C overexpression reduces transgene expression mediated by SeVdp vectors.

### SeV C overexpression inhibits SeVdp RNA synthesis

**Fig 3:**
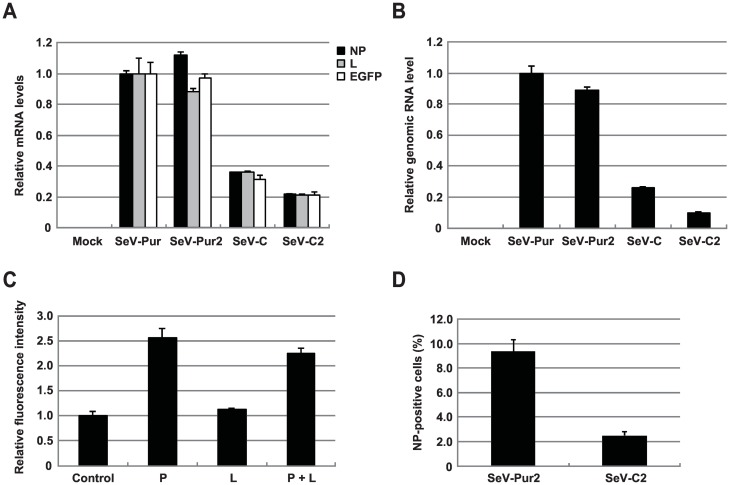
Effects of SeVdp-mediated C overexpression on SeVdp RNA synthesis. (A) Inhibition of SeVdp mRNA synthesis by SeV C overexpression. The *NP*, *L*, and *EGFP* mRNA levels were determined by RT-qPCR. The mRNA levels of SeV-Pur cells were set to 1.0, and relative mRNA levels of all infected cells are indicated. As controls, the mRNA levels in non-infected cells (mock) were also determined. *GAPDH* expression was used to normalize the data. The means and SD (n = 3) are presented. (B) Inhibition of SeVdp genomic RNA synthesis by SeV C overexpression. The levels of genomic RNA were determined by RT-qPCR. The genomic RNA level of SeV-Pur cells was set to 1.0, and relative levels of the infected cells are indicated. Non-infected cells (mock) were used as a negative control. *GAPDH* expression was used to normalize the data. The means and SD (n = 3) are presented. (C) Reversal effect of P expression on C-mediated inhibition. SeV-C2 cells were transfected with an empty plasmid (control), either P or L expression plasmid (P or L), or both P and L expression plasmids (P + L). Two days after transfection, EGFP expression was measured by flow cytometry. The EGFP intensity of cells transfected with the empty plasmid was set to 1.0, and relative fluorescence intensities of all cells are indicated. The means and SD from three replicate experiments are presented. (D) Effects of siRNA against the *L* gene on elimination of SeVdp vectors from infected cells. SeV-Pur2 and SeV-C2 cells were transfected with siRNA against the *L* gene (siL527) or negative control siRNA (siNEG) on days 0, and 6, and NP-positive cells were determined by flow cytometry on day 13. NP-positive cells transfected with the siNEG were set to 100% (data not shown), and the percentages of NP-positive cells transfected with siL527 are indicated. The means and SD from three replicate experiments are presented.

Next, we examined the effects of SeV C overexpression on SeVdp mRNA and genomic RNA synthesis. Total RNA was isolated from SeV-Pur, SeV-Pur2, SeV-C, or SeV-C2 cells and the expression of the *NP*, *P*/*C*/*V*, *L*, and *EGFP* mRNAs was monitored by quantitative reverse transcription PCR (RT-qPCR). All mRNA levels were significantly reduced in SeV-C and SeV-C2 cells compared to those in SeV-Pur and SeV-Pur2 cells (Fig 3A and S4A Fig). Notably, SeV-C2 cells showed lower levels of *EGFP* mRNA than SeV-C cells, which is consistent with the results obtained by flow cytometry analysis (Fig 2B). Western blot analysis also indicated a significant reduction in the level of NP protein in SeV-C2 cells (S5 Fig). These results suggest that C overexpression inhibits SeVdp mRNA synthesis, causing concomitant reductions in the levels of cognate proteins. We confirmed that *C-opt* mRNA was present in SeV-C and SeV-C2 cells, whereas SeV-Pur and SeV-Pur2 cells produced the *Puro*^*r*^ mRNA as expected (S4B Fig).

To examine whether C overexpression affects genomic RNA synthesis, we performed RT-qPCR using the primer set designed to amplify the region corresponding to the *EGFP* gene. Reverse transcription with the sense primer for the *EGFP* gene allows cDNA synthesis from (-)-strand genomic RNA, but not (+)-strand antigenomic RNA and mRNA. Therefore, subsequent PCR can detect only genomic RNA using this procedure. Strikingly, the levels of genomic RNA in SeV-C and SeV-C2 cells were markedly reduced as compared to those in SeV-Pur and SeV-Pur2 cells (Fig 3B), suggesting that SeV C overexpression efficiently inhibited not only mRNA synthesis but also genomic RNA synthesis in the SeVdp system.

A previous study demonstrated that the ability of C proteins to inhibit SeV RNA synthesis depends on coexpression of the P and L proteins [24]. Interestingly, the overexpression of P protein, but not that of L protein, appeared to confer a reversal effect on C-mediated inhibition [24, 26]. To examine whether the expression of P or L could relieve the C inhibitory effect, SeV-C2 cells were transfected with the P or L expression plasmid and EGFP expression was measured by flow cytometry 2 days after transfection. Consistent with the results of previous studies, overexpression of the P significantly reversed EGFP intensity up to 2.5-fold compared to cells transfected with the empty plasmid, whereas L overexpression had no effect (Fig 3C). Cotransfection of the P and L plasmids did not enhance the reversal effect. These results suggest that high levels of P protein against the C protein alleviate the C inhibitory effect in the SeVdp system.

We previously demonstrated that the SeVdp genome could be eliminated from infected cells by blocking SeVdp RNA synthesis using siRNA against the *L* gene (siL527) [10]. The SeV RNA genome replicates in infected cells without chromosomal insertion and serves as a template for mRNA synthesis. Thus, complete deletion of the SeVdp genome abolishes transgene expression. This characteristic is highly advantageous for developing transgene-free iPS cells, which are required for medical applications [10]. Given that excess SeV C protein inhibits SeVdp genomic RNA synthesis, we speculated that SeV C overexpression would facilitate elimination of the SeVdp genome from infected cells. To evaluate this hypothesis, SeV-Pur2 or SeV-C2 cells were repeatedly transfected with siL527 (days 0, and 6), and SeV NP antigen-positive cells were measured by flow cytometry 13 days after the first transfection. As shown in Fig 3D, NP-antigen positive SeV-C2 cells accounted for only 2.5% of cell, whereas 9.4% of SeV-Pur2 cells remained NP-positive. Notably, SeV-Pur2 and SeV-C2 cells showed no adverse effects on proliferation (S6 Fig).

### Regulation of transgene expression by combination of SeV C and endogenous miRNAs

**Fig 4:**
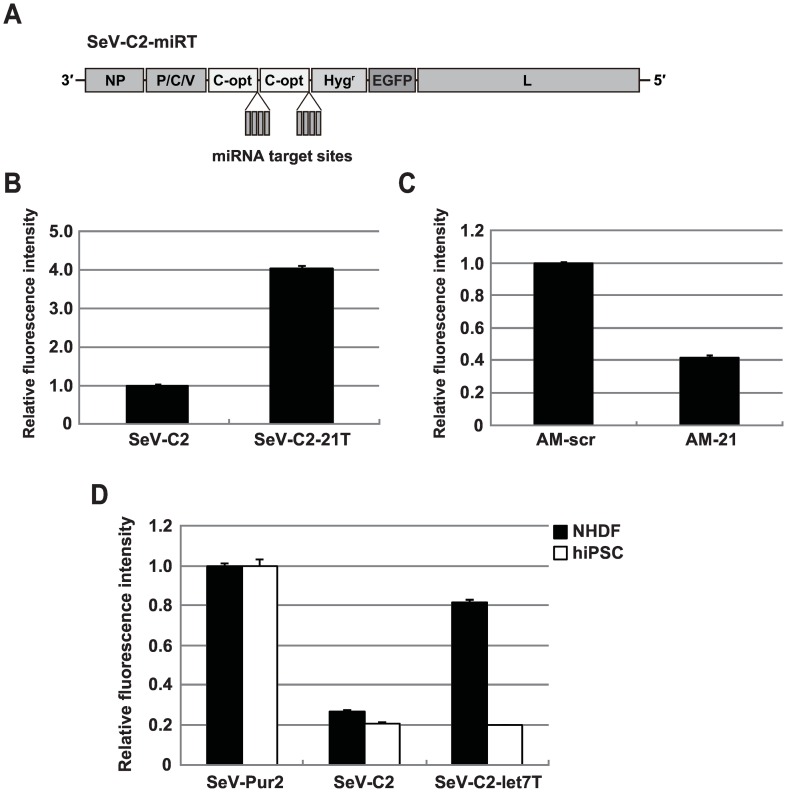
Regulation of transgene expression by endogenous miRNAs. (A) Genome structure of the SeVdp vector containing miRNA target sequences. Four copies of miRNA target sequence were incorporated into the 3′ UTR of both *C-opt* genes on the SeV-C2 backbone (SeV-C2-miRT). To construct SeV-C2-21T and SeV-C2-let7T, miR-21 and let-7a target sequences were incorporated into SeV-C2, respectively. (B) Effect of miR-21 on transgene expression mediated by SeVdp vectors. The EGFP expression levels of SeV-C2 cells and SeV-C2-21T cells were measured by flow cytometry. The EGFP intensity of SeV-C2 cells was set to 1.0, and relative intensity of SeV-C2-21T cells is indicated. The means and SD from three replicate experiments are presented. (C) Effect of antimiR-21 on transgene expression mediated by the SeVdp vector. SeV-C2-21T cells were transfected with 50 nM LNA scramble (AM-scr) or antimiR-21 (AM-21), and EGFP expression levels were measured by flow cytometry. The EGFP intensity of cells transfected with the AM-scr was set to 1.0, and relative intensity of cells transfected with AM-21 is indicated. The means and SD from three replicate experiments are presented. (D) Effect of let-7 on transgene expression mediated by SeVdp vectors. Normal human dermal fibroblasts (NHDF) or human iPS cells (hiPSC) were infected with SeV-Pur2, SeV-C2, or SeV-C2-let7T, and EGFP expression levels were measured by flow cytometry. The EGFP intensities of cells infected with SeV-Pur2 were set to 1.0, and relative fluorescence intensities of SeV-C2 and SeV-C2-let7T cells are indicated. The means and SD from three replicate experiments are presented.

To control transgene expression, we attempted to regulate the SeV C expression using endogenous miRNAs. miRNAs have been successfully exploited to regulate transgene expression in various types of vectors. Thus, we assumed that miRNA-regulated SeV C expression plays a role in controlling transgene expression in target cells. Because miR-21 is reportedly highly abundant in HeLa cells [39], we examined whether miR-21 could be exploited to regulate SeV C expression. To investigate miR-21 activity in HeLa cells, we constructed the psiCHECK reporter plasmid (psi-miR-21) containing one copy of the miR-21 target sequence at the 3′ UTR of the *Reniila* luciferase gene. HeLa S3 cells were transfected with psi-miR-21 or psi-miR-scr, which contains a scramble target sequence, and luciferase activities were measured 24 h after transfection. miR-21 strongly reduced luciferase activity from the psi-miR-21 (S7A Fig). Next, we constructed the SeVdp vector, named as SeV-C2-21T, which contains four copies of the miR-21 target sequence at the 3′ UTR of *C-opt* genes on the SeV-C2 vector backbone (Fig 4A). HeLa S3 cells were infected with SeV-C2 or SeV-C2-21T, and EGFP expression was measured by flow cytometry after selection with hygromycin B. SeV-C2-21T cells increased the EGFP expression by up to 4-fold as compared to SeV-C2 cells (Fig 4B). To block miR-21 activity, the miR-21 seed-targeting 8-mer locked nucleic acid (LNA) oligonucleotide (antimiR-21) was transfected into SeV-C2-21T cells. The antimiR-21 decreased EGFP expression levels (Fig 4C). These results suggest that miRNA-mediated SeV C modulation regulates transgene expression mediated by the SeVdp vector.

Next, we attempted to control transgene expression depending on cell-type specific miRNA activity. Previous studies demonstrated that human fibroblasts express a large amount of let-7, whereas the expression was lowered in human pluripotent stem cells [40, 41]. We examined let-7 activity in NHDF and hiPS cells in a psiCHECK reporter assay and found that strong gene silencing occurred only in NHDF cells (S7B Fig). Thus, we constructed an SeVdp vector, SeV-C2-let7T, which contains four copies of the let-7a target sequence at the 3′ UTR of *C-opt* genes (Fig 4A). NHDF and hiPS cells were infected with SeV-Pur2, SeV-C2, or SeV-C2-let7T and EGFP expression was measured by flow cytometry. Although both NHDF and hiPS cells harboring the SeV-C2 showed significantly reduced EGFP expression compared to that in cells harboring SeV-Pur2, SeV-C2-let7T reduced EGFP expression in hiPS cells but not in NHDF cells (Fig 4D). These results strongly suggest that SeVdp-mediated transgene expression can be controlled in target cells by modulating the SeV C expression using cell-type specific miRNA.

## Discussion

Cytoplasmic negative-strand RNA viruses have been widely used as oncolytic and vaccine vectors [42, 43]. Generally, these types of viruses strongly induce an innate immune response by activating interferon signaling pathways [44]. Because of this potential cytotoxicity, most cytoplasmic RNA viruses are unable to establish persistent infection in mammalian cells. However, we previously demonstrated that SeV, a cytoplasmic negative-strand RNA virus, can be used as a persistent and stable gene expression vector [11]. We further showed that a novel SeV vector, SeVdp, lacking all structural genes dispensable for viral transcription and replication, allowed long-term expression of multiple transgenes in cultured cells [10].

In the present study, we proposed a unique strategy for regulating transgene expression mediated by SeVdp vectors. Prolonged expression of a transgene in non-target cells may limit cell growth and viability. Thus, controlling transgene expression is very important for reducing negative effects. We found that SeV C overexpression significantly reduced SeVdp-mediated transgene expression (Fig 2B). This effect would be attributed to the inhibition of SeVdp RNA synthesis (Fig 3A and 3B and S4A Fig). Importantly, modulation of SeV C expression by endogenous miRNA played a role in controlling transgene expression in a cell-specific manner (Fig 4D). There have been few reports regarding systems for regulating gene expression using negative-strand RNA virus platforms [45, 46]. A recent study demonstrated that a ligand-responsive ribozyme could be used to regulate glycoprotein expression in an engineered measles virus, inhibiting viral propagation in the presence of the ligand [46]. Apart from previously reported systems using regulatory RNAs, to the best of our knowledge, we demonstrate for the first time that a viral nonstructural protein can be used as an effective regulator for cytoplasmic RNA vectors.

To achieve a high level of SeV C expression, we constructed the SeVdp vectors, SeV-C and SeV-C2, which contain human codon-optimized C (*C-opt*) gene(s) (Fig 2A). The SeVdp vectors used in this study possess an intact *P* gene, which produces not only P protein but also four wild-type C proteins. Thus, all of SeVdp vectors, including SeV-Pur and SeV-Pur2, express steady-state levels of C proteins in infected cells. A previous study suggested that SeV lacking *C* genes caused aberrant viral RNA synthesis, resulting in the production of predominantly non-infectious virions containing antigenomic RNA [47]. Thus, it is likely that the steady-state level of C proteins is necessary for normal vector production and recovery from the packaging cells. Notably, we have not yet succeeded in recovery of an SeVdp vector with all *C* genes deleted, although SeVdp vectors used in this study were recovered as high titers of infectious viral stocks (10^6^–10^7^ cell-infectious units/mL), suggesting that our strategy had no serious adverse effects on SeVdp vector production.

SeV C overexpression reduced the levels of SeVdp mRNA and genomic RNA (Fig 3A and 3B), although the level of antigenomic RNA could not be correctly determined by RT-qPCR because of a technical difficulty in distinguishing antigenomic RNA and SeVdp mRNAs. Curran *et al*. initially postulated that SeV C proteins were responsible for inhibiting viral mRNA synthesis rather than genome replication [24]. They later suggested that C proteins also inhibited SeV replication in a promoter-specific manner [25]; however, a large excess of C proteins appears to inhibit both genome and antigenome synthesis. [26]. In our system, it is likely that SeV-C and SeV-C2 cells produced adequate amounts of C proteins to inhibit both transcription and genome synthesis. Importantly, we showed that P overexpression effectively reversed the C inhibitory effect in SeV-C2 cells (Fig 3C). According to previous reports, the ratio of P to C is important for determining the C inhibitory effect [24, 26], although the P and L proteins are components of the SeV RdRp complex. The precise mechanism of how P protein alleviates the C inhibitory effect remains unclear, as C proteins were suggested to directly interact with the L protein, but not the P protein, inhibiting viral RNA synthesis [48, 49]. However, our findings suggest that concurrently modulating P and C expression might regulate transgene expression more efficiently in the SeVdp system. In future studies, a refined system for controlling P and C expression simultaneously should be constructed.

The miRNA regulatory system has been successfully exploited to control transgene expression in various types of vectors. Robust activity and tissue-specific expression signatures in various miRNAs are broadly applicable for controlling gene expression in target cells. We exploited endogenous miRNA activities to regulate SeV C expression. We found that inhibition of C-opt expression by miR-21 markedly increased transgene expression in HeLa S3 cells (Fig 4B). Importantly, transfection of the inhibitor for miR-21 decreased transgene expression (Fig 4C), indicating that transgene expression is reversibly regulated depending on miRNA activity. We further demonstrated that transgene expression could be differentially controlled by modulating SeV C expression with let-7 in NHDF and hiPS cells (Fig 4D). Let-7 family members exhibit high sequence similarity [50] and contain a conserved seed sequence, corresponding to nucleotide positions 2–7 from the 5′ end, which are responsible for binding specificity [51]. Most, if not all, let-7 family members are thought to participate in binding to the let-7a target sequence and strongly inhibit C-opt expression in NHDF cells.

The SeVdp vector containing the *Klf4*, *Oct4*, *Sox2*, and *c-Myc* genes (SeVdp(KOSM)) efficiently reprogrammed human fibroblasts to hiPS cells [52]. In this study, we showed that the effect of siL527 on elimination of the SeVdp vector was much stronger in SeV-C2 cells than in SeV-Pur2 cells (Fig 3D), suggesting that a decreased level of genomic RNA is advantageous for generating transgene-free cells. Thus, the use of C proteins in combination with let-7 to regulate SeVdp-mediated transgene expression may be applicable for generating iPS cells from fibroblasts. The SeVdp(KOSM) equipped with the C-based regulatory system is thought to maintain efficient Klf4, Oct4, Sox2, and c-Myc expression in NHDF cells (let-7 ON state), but in turn decreases the expression in hiPS cells (let-7 OFF state). This vector may reduce cytotoxicity and facilitate the elimination of the SeVdp(KOSM) genome from hiPS cells; further analyses are required to determine this. Currently, SeV vectors have received increasing attention as potent tools for advanced cell reprogramming and stem cell research. The regulation of SeV-mediated transgene expression is highly desired to provide efficacy and safety. Further refinement of our strategy will facilitate the development of an SeVdp vector capable of precisely controlling transgene expression.

## Supporting Information

S1 Fig Comparison of EGFP expression mediated by SeVdp vectors. (A) HeLa S3 cells were infected with SeV-Pur, SeV-Pur2, SeV-C, or SeV-C2, and treated with hygromycin B. EGFP expression was detected by fluorescence microscopy. Phase contrast images are also presented. (B) EGFP expression levels were measured by flow cytometry and the data is shown as histograms.
(TIF)

S2 Fig Effects of SeV C overexpression on transgene expression in various types of cells. (A) HEK293 or LLC-MK_2_ cells were infected with either SeV-Pur2 or SeV-C2 and treated with hygromycin B. EGFP expression levels were measured by flow cytometry. The fluorescence intensities of SeV-Pur2 cells were set to 1.0 and the relative intensities of SeV-C2 cells are indicated. The means and SD from three replicate experiments are presented. (B) Genomes of SeV-C2-Luc and SeV-Pur2-Luc contain firefly luciferase gene (Luc) rather than the *EGFP* gene. BHK/T7/151M(SE) cells harboring SeV-Pur2-Luc or SeV-C2-Luc were lysed and luciferase activity was measured. The luciferase activity of SeV-Pur2-Luc cells was set to 1.0 and the relative activity of SeV-C2-Luc cells is indicated. The means and SD from three replicate experiments are presented.
(TIF)

S3 Fig Efficacy of the siRNA against human codon-optimized C gene. (A) HeLa S3 cells were co-transfected with the HaloTag-fused C-opt expression plasmid and negative control siRNA (siNEG) or siRNA against the *C-opt* (siC-opt). Two days after transfection, the cells were stained with the HaloTag TMR ligand and DAPI. (B) HaloTag-fused C-opt protein levels were determined by western blot analysis. ß-Actin levels were determined as an internal control.
(TIF)

S4 Fig Quantitative RT-PCR analyses of SeVdp mRNAs. (A) *P*/*C*/*V* mRNA levels were determined by RT-qPCR. The mRNA level of SeV-Pur cells was set to 1.0 and the relative mRNA levels of all infected cells are indicated. Non-infected cells (mock) were used as a negative control. *GAPDH* expression was used to normalize the data. The means and SD (n = 3) are presented. (B) The *Puro*^*r*^ or *C-opt* mRNA levels of HeLa S3 cells harboring the SeV-Pur, SeV-Pur2, SeV-C, or SeV-C2 were determined by RT-qPCR. As a control, the mRNA level in non-infected cells (mock) was also determined. The *Puro*^*r*^ mRNA level in SeV-Pur cells (upper) or the *C-opt* mRNA level in SeV-C cells (bottom) was set to 1.0, and the relative mRNA levels are indicated. *GAPDH* expression was used to normalize the data. The means and SD (n = 3) are presented.
(TIF)

S5 Fig Comparison of NP protein expression mediated by SeVdp vectors. Protein samples were extracted from non-infected HeLa S3 cells (mock) or cells harboring the SeVdp vector as indicated, and levels of the NP protein were determined by western blot analysis. ß-Actin protein levels were determined as an internal control.
(TIF)

S6 Fig Cell proliferation analysis. The proliferation of HeLa S3 cells harboring either SeV-Pur2 or SeV-C2 was measured using the MTT assay on the day of cell seeding (day 0) and at 1, 2, 3, 4, and 6 days after seeding. Non-infected cells (mock) were used as controls. The means and SD from four replicate experiments are presented.
(TIF)

S7 Fig Effects of endogenous miRNAs on interfering with the reporter gene expression. (A) The reporter construct, which contains one copy of the miR-21 target sequence (psi-miR-21) or that of a scramble target sequence (psi-miR-scr) at the 3′ UTR of the *Renilla* luciferase gene in the psiCHECK-2 plasmid, was transfected into HeLa S3 cells and luciferase activities were determined 24 h after transfection. Luciferase activity determined from the cells transfected with psi-miR-scr was set to 100%. The means and SD from three replicate experiments are presented. (B) The psiCEHCK reporter construct containing one copy of the let-7a target sequence (psi-let-7a) or the psi-miR-scr was transfected into human dermal fibroblasts (NHDF) or human iPS cells (hiPSC). Luciferase activities were determined 24 h after transfection. The luciferase activities determined from the cells transfected with psi-miR-scr were set to 100%. The means and SD from three replicate experiments are presented.
(TIF)
