# Supplementary figures and images for: Novel Strategy to Control Transgene Expression Mediated by a Sendai Virus-Based Vector Using a Nonstructural C Protein and Endogenous MicroRNAs
Source: PLoS One. 2016 Oct 20;11(10):e0164720. doi: 10.1371/journal.pone.0164720 (PMC5072705; doi:10.1371/journal.pone.0164720)

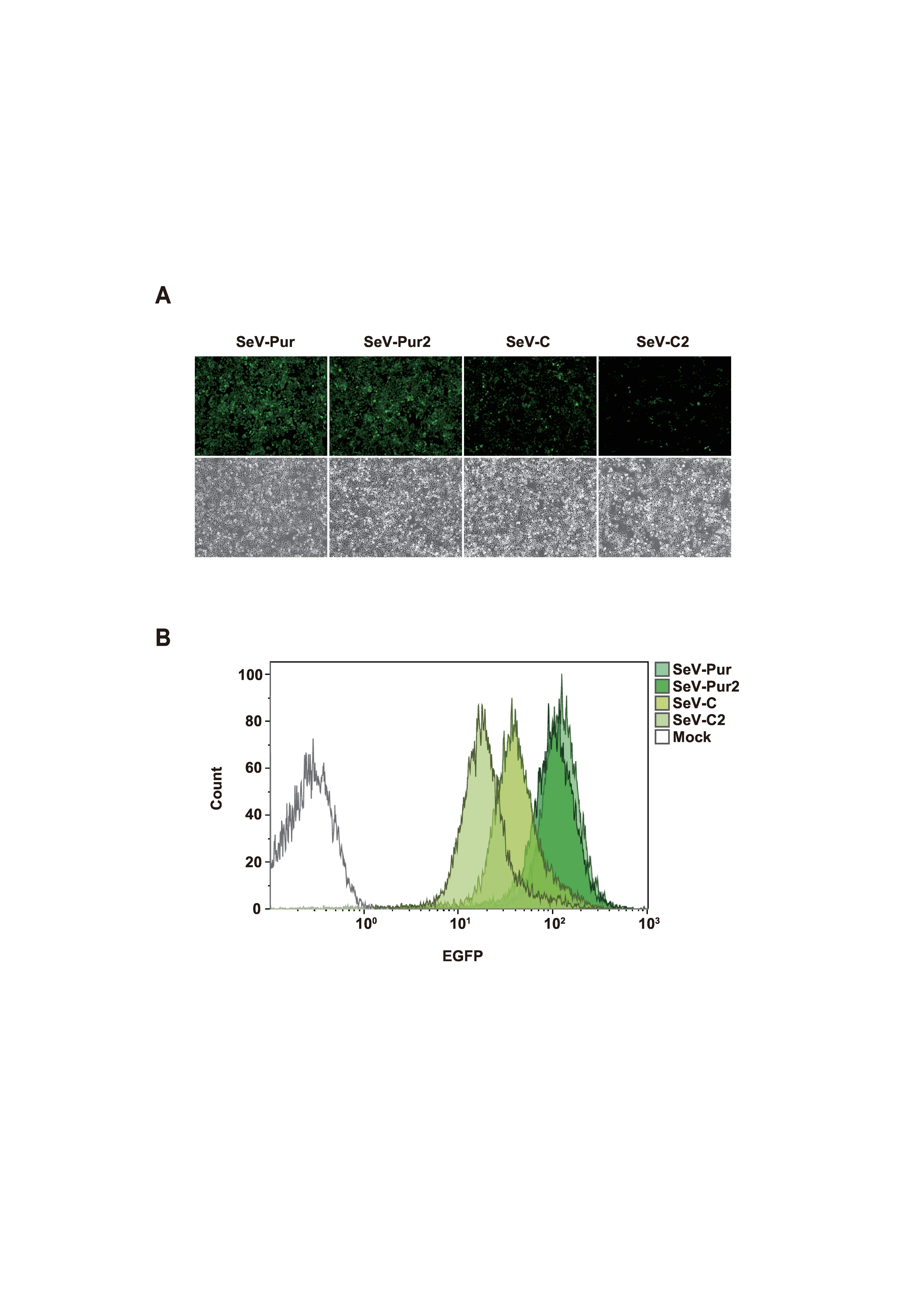

Supplement: S1 Fig — (A) HeLa S3 cells were infected with SeV-Pur, SeV-Pur2, SeV-C, or SeV-C2, and treated with hygromycin B. EGFP expression was detected by fluorescence microscopy. Phase contrast images are also presented. (B) EGFP expression levels were measured by flow cytometry and the data is shown as histograms. (TIF) [file pone.0164720.s001.tif]

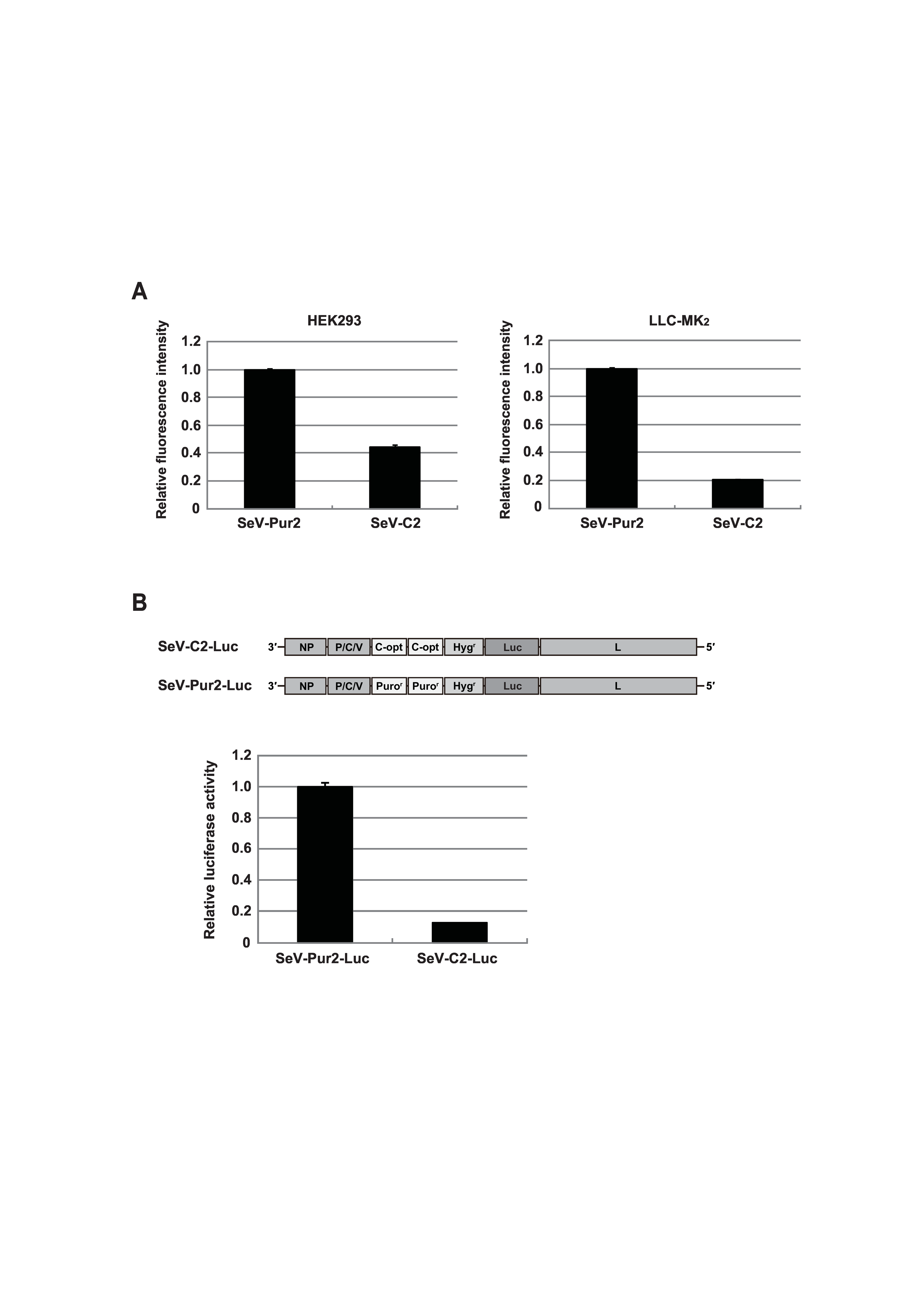

Supplement: S2 Fig — (A) HEK293 or LLC-MK2 cells were infected with either SeV-Pur2 or SeV-C2 and treated with hygromycin B. EGFP expression levels were measured by flow cytometry. The fluorescence intensities of SeV-Pur2 cells were set to 1.0 and the relative intensities of SeV-C2 cells are indicated. The means and SD from three replicate experiments are presented. (B) Genomes of SeV-C2-Luc and SeV-Pur2-Luc contain firefly luciferase gene (Luc) rather than the EGFP gene. BHK/T7/151M(SE) cells harboring SeV-Pur2-Luc or SeV-C2-Luc were lysed and luciferase activity was measured. The luciferase activity of SeV-Pur2-Luc cells was set to 1.0 and the relative activity of SeV-C2-Luc cells is indicated. The means and SD from three replicate experiments are presented. (TIF) [file pone.0164720.s002.tif]

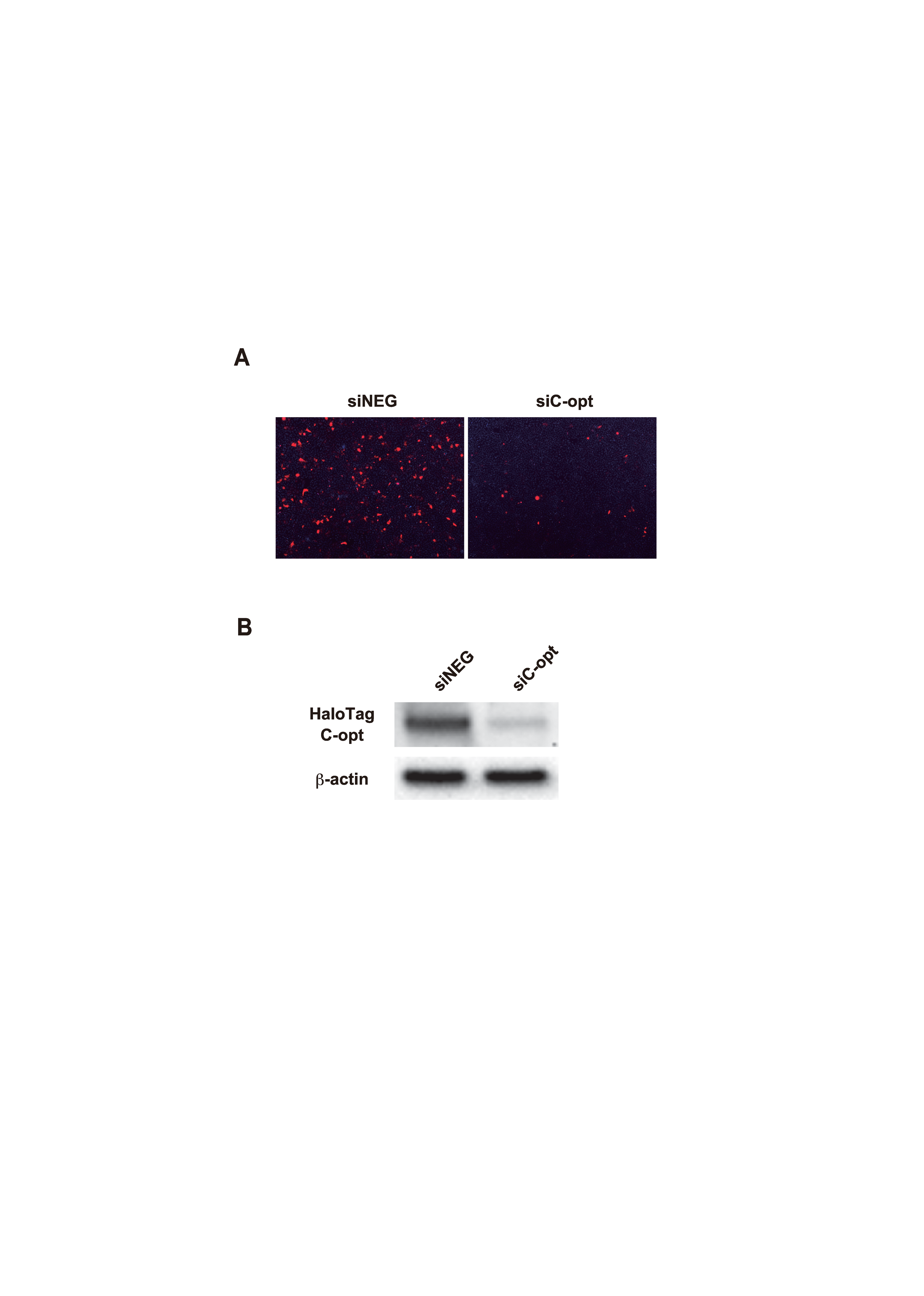

Supplement: S3 Fig — (A) HeLa S3 cells were co-transfected with the HaloTag-fused C-opt expression plasmid and negative control siRNA (siNEG) or siRNA against the C-opt (siC-opt). Two days after transfection, the cells were stained with the HaloTag TMR ligand and DAPI. (B) HaloTag-fused C-opt protein levels were determined by western blot analysis. ß-Actin levels were determined as an internal control. (TIF) [file pone.0164720.s003.tif]

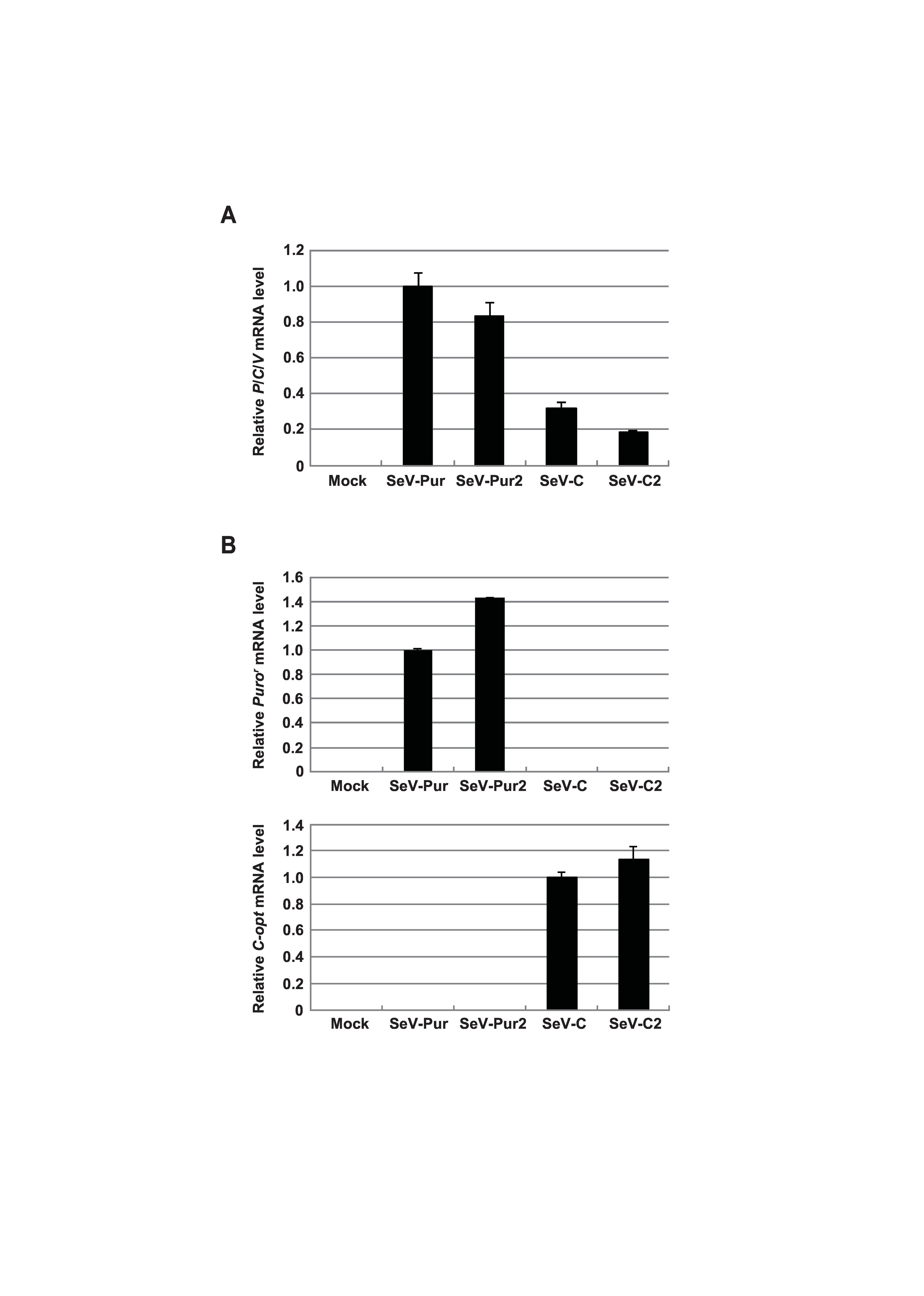

Supplement: S4 Fig — (A) P/C/V mRNA levels were determined by RT-qPCR. The mRNA level of SeV-Pur cells was set to 1.0 and the relative mRNA levels of all infected cells are indicated. Non-infected cells (mock) were used as a negative control. GAPDH expression was used to normalize the data. The means and SD (n = 3) are presented. (B) The Puror or C-opt mRNA levels of HeLa S3 cells harboring the SeV-Pur, SeV-Pur2, SeV-C, or SeV-C2 were determined by RT-qPCR. As a control, the mRNA level in non-infected cells (mock) was also determined. The Puror mRNA level in SeV-Pur cells (upper) or the C-opt mRNA level in SeV-C cells (bottom) was set to 1.0, and the relative mRNA levels are indicated. GAPDH expression was used to normalize the data. The means and SD (n = 3) are presented. (TIF) [file pone.0164720.s004.tif]

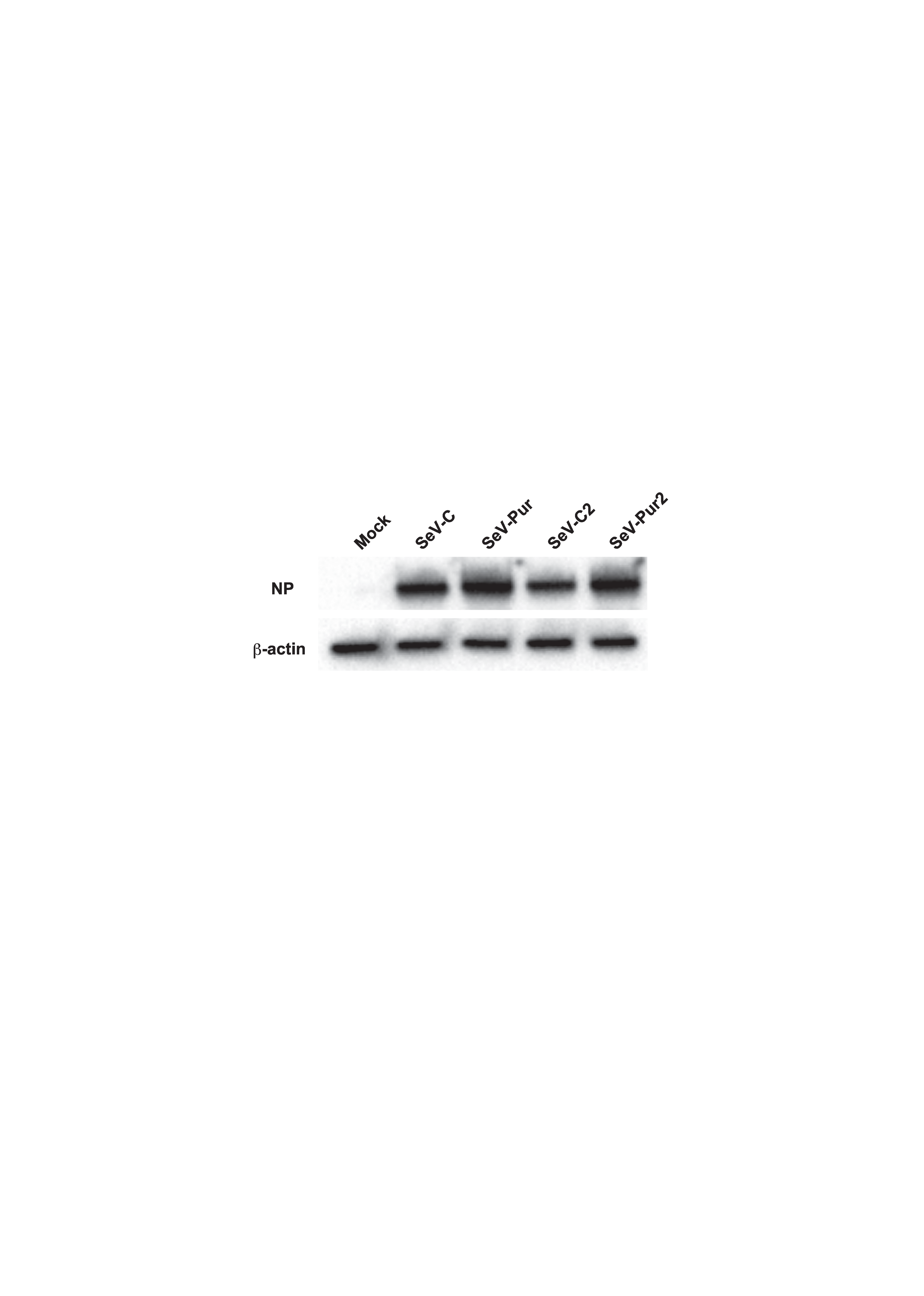

Supplement: S5 Fig — Protein samples were extracted from non-infected HeLa S3 cells (mock) or cells harboring the SeVdp vector as indicated, and levels of the NP protein were determined by western blot analysis. ß-Actin protein levels were determined as an internal control. (TIF) [file pone.0164720.s005.tif]

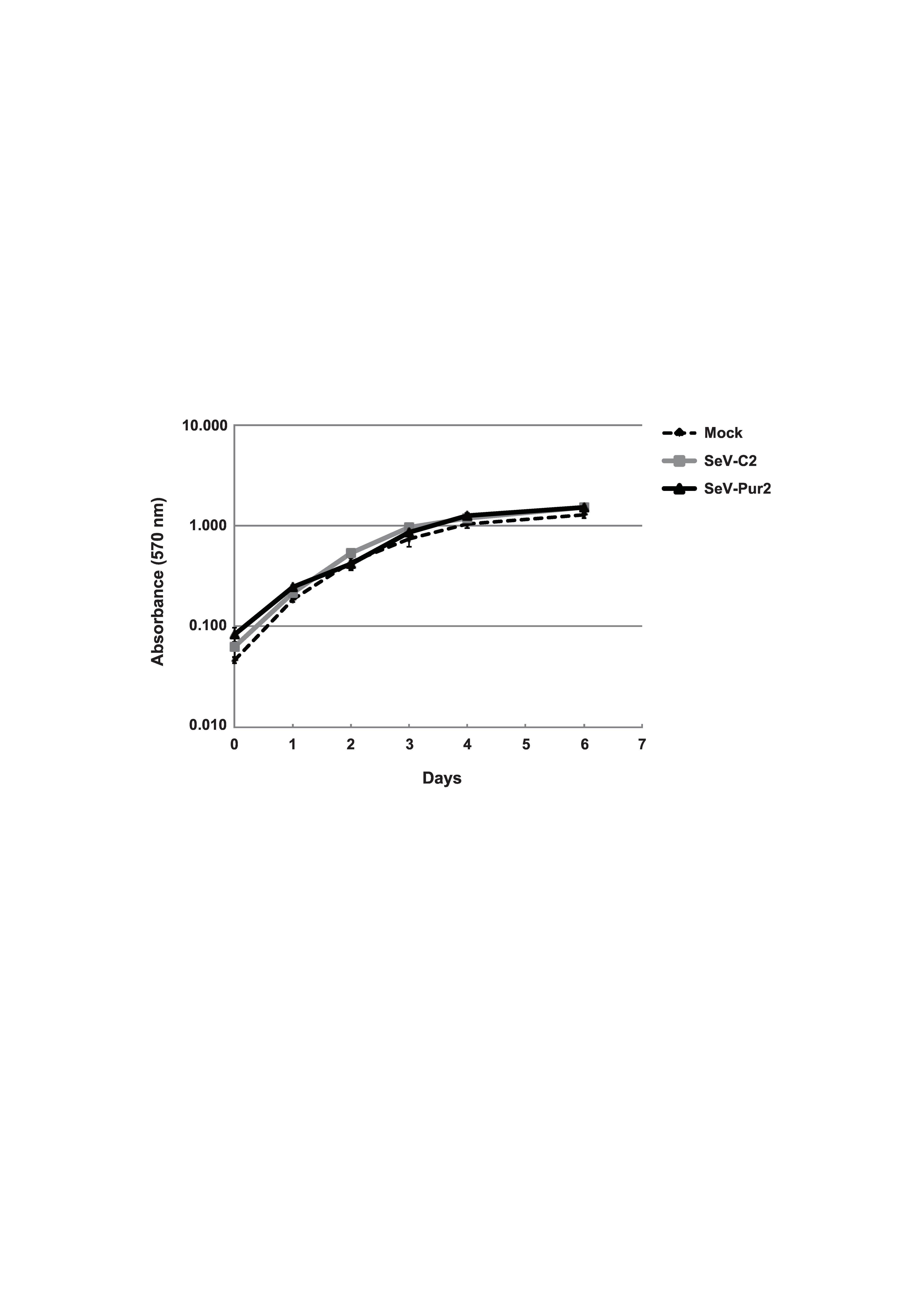

Supplement: S6 Fig — The proliferation of HeLa S3 cells harboring either SeV-Pur2 or SeV-C2 was measured using the MTT assay on the day of cell seeding (day 0) and at 1, 2, 3, 4, and 6 days after seeding. Non-infected cells (mock) were used as controls. The means and SD from four replicate experiments are presented. (TIF) [file pone.0164720.s006.tif]

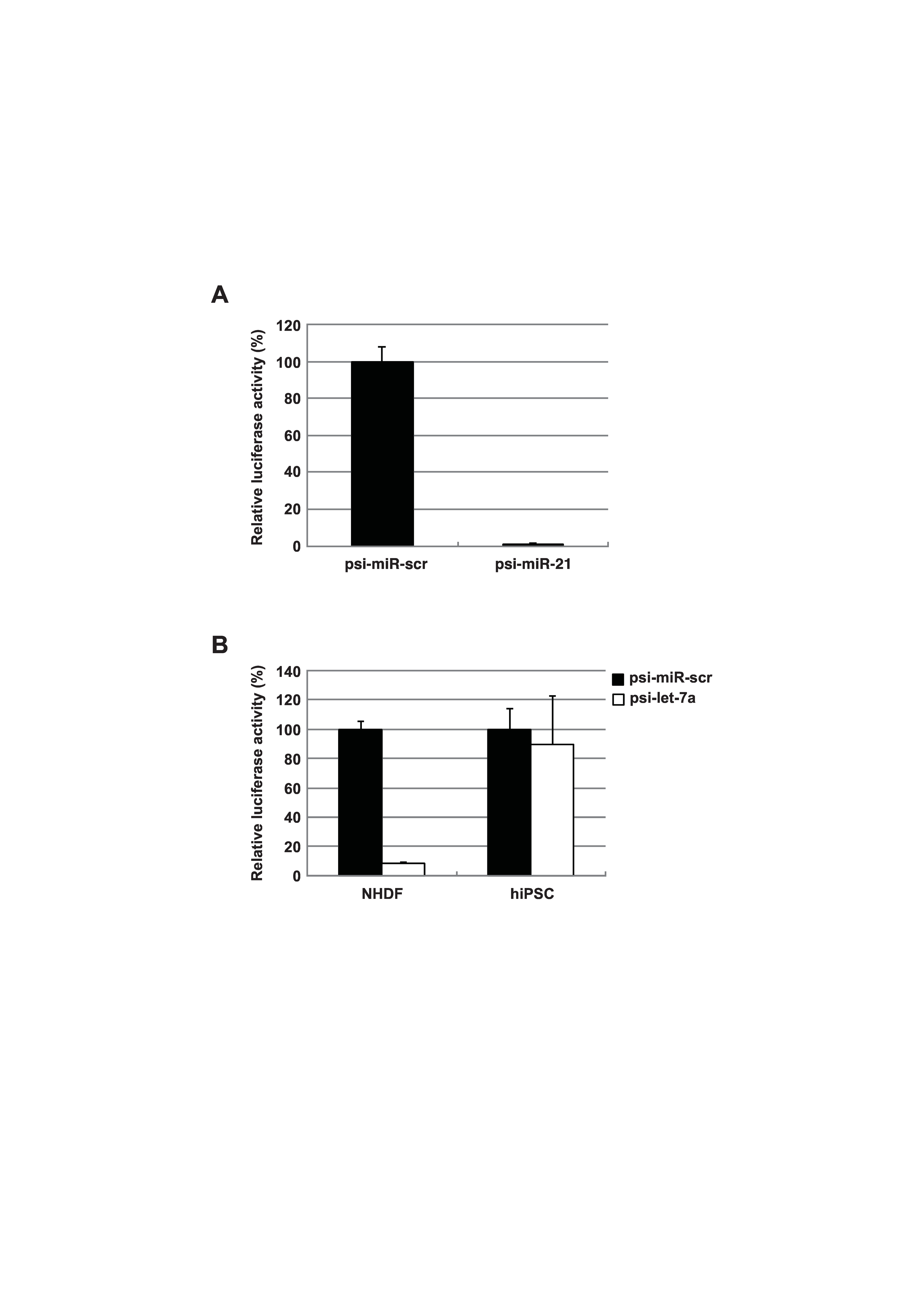

Supplement: S7 Fig — (A) The reporter construct, which contains one copy of the miR-21 target sequence (psi-miR-21) or that of a scramble target sequence (psi-miR-scr) at the 3′ UTR of the Renilla luciferase gene in the psiCHECK-2 plasmid, was transfected into HeLa S3 cells and luciferase activities were determined 24 h after transfection. Luciferase activity determined from the cells transfected with psi-miR-scr was set to 100%. The means and SD from three replicate experiments are presented. (B) The psiCEHCK reporter construct containing one copy of the let-7a target sequence (psi-let-7a) or the psi-miR-scr was transfected into human dermal fibroblasts (NHDF) or human iPS cells (hiPSC). Luciferase activities were determined 24 h after transfection. The luciferase activities determined from the cells transfected with psi-miR-scr were set to 100%. The means and SD from three replicate experiments are presented. (TIF) [file pone.0164720.s007.tif]
